# Assessing Safety Culture in Pharmacies: The psychometric validation of the Safety Attitudes Questionnaire (SAQ) in a national sample of community pharmacies in Sweden

**DOI:** 10.1186/1472-6904-10-8

**Published:** 2010-04-11

**Authors:** Annika Nordén-Hägg, J Bryan Sexton, Sofia Kälvemark-Sporrong, Lena Ring, Åsa Kettis-Lindblad

**Affiliations:** 1 Department of Pharmacy, Uppsala University, Box 570, S-751 23 Uppsala, Sweden; 2 The Johns Hopkins University, School of Medicine, Quality & Safety Research Group, 1909 Thames Street, 2nd floor, Baltimore, Maryland, 21231, USA; 3 Department of Public Health and Caring Services, Uppsala University, Box 564, S-751 23 Uppsala, Sweden; 4 Health Economics & Outcomes Research, R&D, AstraZeneca, S-151 85 Södertälje, Sweden

## Abstract

**Background:**

Safety culture assessment is increasingly recognized as an important component in healthcare quality improvement, also in pharmacies. One of the most commonly used and rigorously validated tools to measure safety culture is the Safety Attitudes Questionnaire; SAQ. This study presents the validation of the SAQ for use in Swedish pharmacies. The psychometric properties of the translated questionnaire are presented

**Methods:**

The original English language version of the SAQ was translated and adapted to the Swedish context and distributed by e-mail. The survey was carried out on a national basis, covering all 870 Swedish community pharmacies. In total, 7,244 questionnaires were distributed. Scale psychometrics were analysed using Cronbach alphas and intercorrelations among the scales. Multiple group confirmatory factor analysis (CFA) was conducted.

**Results:**

SAQ data from 828 community pharmacies in Sweden, including 4,090 (60.22%) pharmacy personnel out of 6,683 eligible respondents, were received. There were 252 (28.97%) pharmacies that met the inclusion criteria of having at least 5 respondents and a minimum response rate of 60% within that pharmacy.

The coefficient alpha value for each of the SAQ scales ranged from .72 to .89. The internal consistency results, in conjunction with the confirmatory factor analysis results, demonstrate that the Swedish translation of the SAQ has acceptable to good psychometric properties. Perceptions of the pharmacy (Teamwork Climate, Job Satisfaction, Perceptions of Management, Safety Climate, and Working Conditions) were moderately to highly correlated with one another whereas attitudes about stress (Stress Recognition) had only low correlations with other factors. Perceptions of management showed the most variability across pharmacies (SD = 26.66), whereas Stress Recognition showed the least (SD = 18.58). There was substantial variability ranging from 0% to 100% in the percent of positive scores for each of the factors across the 252 pharmacies.

**Conclusions:**

The Swedish translation of the SAQ demonstrates acceptable construct validity, for capturing the frontline perspective of safety culture of community pharmacy staff. The psychometric results reported here met or exceeded standard guidelines, which is consistent with previous studies using the SAQ in other healthcare settings and other languages.

## Background

The occurrence of dispensing errors in community pharmacies might be a marker for patient safety in the operational units. Assessment of this variable in pharmacies is however limited. Other indicators that evaluate safety-related norms and behaviours, i.e. safety culture, are increasingly being used across different disciplines and industries. These metrics of safety have gained increasing interest as a fundamental and important factor in health-care [1,2], as they impact both patient outcomes and health care costs [1,3]. Influential organizations within the health field like the WHO [4], National Patient Safety Foundation [5], the Joint Commission [6] as well as the Institute for Health care Improvement [7], are thus promoting the concept of safety culture.

Variations on the definition of safety culture exist. The one provided by The Advisory Committee on the Safety of Nuclear Installations [8] can easily be adapted to the context of patient safety in health care: *"The safety culture of an organization is the product of individual and group values, attitudes, perceptions, competencies, and patterns of behaviour that determine the commitment to, and the style and proficiency of, an organization's health and safety management*." Pharmacies are an important and well-integrated part of the health system and the definition of safety culture is applicable also on these settings.

Measuring and understanding the safety culture of an organization is becoming an important diagnostic tool when trying to assess the quality of care provided. Methods designed to measure safety culture within health care systems have a common feature in that they predominantly use surveys to assess individual attitudes covering areas related to work environment, adherence to guidelines and safety concerns [9,10]. One of the differences is the target group, where some surveys encompass all the staff in one working site and thereby elicit a snapshot of the safety climate in that specific setting, while others focus on the managerial view of safety within an organization [11].

The assessment of safety culture in pharmacies has recently begun to develop and the consistency of methods and instruments used across pharmacies needs to be further elaborated. Hospital pharmacies have been included in overall hospital based safety culture assessments [12-15]. These results have only been reported on an aggregated level, and as of yet we are not aware of safety culture results across hospital pharmacies in the peer reviewed literature. A few studies describe the development of survey instruments to assess the safety culture in community pharmacies. In UK the Manchester Patient Safety Assessment has been developed for use within primary care [16] and is also adapted for use in community pharmacies [17]. This instrument allows the staff to self-report their level of safety culture maturity. Another way to measure safety culture was recently established by Ashcroft and Parker, by a questionnaire directed towards pharmacists working within community pharmacies [18]. Yet another survey tool is used by the Institute for Safe Medication Practice (ISMP) within different institutions including community pharmacies, to assess the medication safety practices, providing the possibility to compare the result of one organization to the aggregate experience of similar organizations [19]. Despite the existence of these instruments, no studies to date, to our knowledge, have been published in which the safety culture in community pharmacies has been assessed.

One of the most commonly used tools to measure safety culture across health care settings, is the validated Safety Attitudes Questionnaire; SAQ [9,20]. The SAQ has been used to explore the relationship between safety culture in health care and patient outcomes and has been shown to correlate with fewer medication errors, shorter lengths of stay, and fewer adverse outcomes [9,21,22]. It has been used within community pharmacies (unpublished data, JB Sexton) and has been established to be just as feasible in these settings as in other parts of the health care system. The generic nature of the SAQ allows for useful comparisons across the health care sector.

The quality of the services and the safety of people served by the pharmacies are usually regulated by a framework of laws, which establish the minimum requirements. In addition, proprietors and managers of pharmacies often use internal procedures and guidelines. To ensure that these requirements regarding quality and patient safety are maintained, a systematic examination of the safety-related norms and behaviours across pharmacies is needed.

The aim of this study is to adapt the generic SAQ Short Form version, to be used in Swedish community pharmacies to map their safety culture. Psychometric results and benchmarking comparison data are also presented. To our knowledge this is the first safety culture assessment directed to all staff at all community pharmacies in an entire nation.

## Methods

The survey was carried out on a national basis, covering all 870 Swedish community pharmacies. At the time of the study, the National Corporation of Pharmacies in Sweden organised all community pharmacies in Sweden. There were approximately 7,000 staff members within these pharmacies; the largest job category is made up of pharmacists and prescriptionists. In 2007 the community pharmacies dispensed 63,000,000 prescription items [23].

All the people listed as employed in the community pharmacies on December 1^st^, 2007, were invited to participate. In total, 7,244 questionnaires were distributed. The staff consisted of 7% pharmacists (five years university education) and 54% prescriptionists (three years university education). Pharmacists and prescriptionists have equal medication dispensing certification, corresponding to that of an international pharmacist degree. For the purposes of this paper, there are no substantial differences between pharmacists and prescriptionists when it comes to working in Swedish pharmacies. The remaining staff were pharmacy technicians with secondary school education (29%), pharmacy assistants with company training only (1%) and "Others" (9%). Half of the "Other" category was pharmacists, prescriptionists or pharmacy technicians with an additional degree or education (for example nurse assistant).

### Approval of Ethics Committee

No approval was required from the ethics committee according to the Swedish law at the time of the data collection. Ethic considerations were met however; responding to the questionnaire was voluntary and all answers were de-identified to maintain confidentiality.

### The Safety Attitudes Questionnaire; SAQ

The Safety Attitude Questionnaire was developed over 15 years, to assess the quality of safety and teamwork related norms and behaviours of individual workers, in a particular setting [20]. The questionnaire comprises six factors: Teamwork Climate, Safety Climate, Perceptions of Management, Job Satisfaction, Working Conditions, and Stress Recognition and it have been demonstrated to have good psychometric properties. The SAQ has been adapted for use in several different settings, including intensive care units [24,25], operating theatres [26], labor and delivery units [27], emergency departments, ambulatory clinics [28] and pharmacies [29]. In the latter setting hospital pharmacies were included as a part of a hospital wide survey. The questionnaire items are generically framed, changing only references to the setting (e.g. "in this clinic" vs. "in this pharmacy") and role (e.g. "physicians" vs. "pharmacists"). Factor definitions including examples of items included in the different factors, are presented in Table 1. The full version of the questionnaire included 60 items, of which only 30 were scaled [11]; the generic SAQ Short Form version used for hospital-wide administration uses the 30 scaled items. The version serving as a template for translation and adaptation in this study, for use in Swedish Community pharmacies, used 36 items, of which six constituted non-scaled items. Non-scaled items are used in addition to the scaled items so that additional diagnostic and actionable information can be collected during the same survey administration.

**Table 1:** SAQ factor definitions and example items

Factor definition	Example of items included in factor
***Job satisfaction: ***positivity about the work experience	I like my job This pharmacy is a good workplace

***Teamwork climate: ***perceived quality of collaboration between personnel	I receive the support I need from other staff in this pharmacy to help the customers. The staff members at this pharmacy work together as a well-functioning team.

***Safety climate: ***perceptions of a strong and proactive organizational commitment to safety	I would feel safe as a customer here. Dispensing errors are handled in a correct way at this pharmacy.

***Perceptions of management***: approval of managerial action	The management of this pharmacy supports me in my daily work. The management of this pharmacy would not consciously endanger patient safety.

***Stress recognition: ***acknowledgement of how performance is influenced by stressors	I am less efficient at work when I am tired. I am more likely to make mistakes in stressful or unpleasant situations.

***Working conditions: ***perceived quality of the work environment and logistical support (staffing, training, etc.)	This pharmacy does a good job of training new staff members. Newly employed staff is monitored adequately in this pharmacy

### Translation and adaptation of SAQ

The translation of the generic SAQ Short Form version into Swedish was carried out in several steps. This procedure utilized recommended guidelines for translation of patient-reported outcomes instruments [30]. The first translation into Swedish, by an independent translator (pharmacist with Swedish as native language, proficient in English) was discussed within the research group, which included the creator of the original SAQ; JBS. Following consensus of the wording, face validity was established by testing the resulting preliminary Swedish version of the questionnaire on a sample of pharmacy staff (n = 10) with varying education and age. (These staff members were later included in the main survey.) The respondents were asked to comment on their perception of the items in a "think aloud" manner. Based on this procedure, the questionnaire was reformulated. Following this a back-translation was performed by a professional non-pharmacist translator, and again discussed within the research group. In the Additional file 1 the original version of the SAQ, as well as the Swedish translation and the back-translation is available.

A pilot study was subsequently performed to ensure acceptable psychometric properties of the Swedish version of the questionnaire. The survey was administered to 155 pharmacist and prescriptionist students during their pharmacy internship at the end of their training.

### Items and factors

Some amendments and supplementary actions were performed in the Swedish version of the SAQ, as compared to the original version (Table 2). In addition, the negatively worded items "In this pharmacy, it is difficult to speak up if I perceive a problem with patient care" and "In this pharmacy, it is difficult to discuss errors" were supplemented by positively worded versions of the same questions.

**Table 2:** Translation and adaptation of the Swedish SAQ Community pharmacy version

SAQ version			Comments
**Original**	**Swedish Community pharmacy**	**Back-translation of Swedish version**	

***Team work:***			

My input is well received in this clinical area.	Mina synpunkter och förslag - min input - tas väl emot på det här apoteket.	My views and suggestions - my input - is well received in this pharmacy.	The word "input" is sometimes used in Swedish, and it was hence kept but supported by an explanatory Swedish wording.

In this pharmacy, it is difficult to speak up if I perceive a problem with patient care.	Om jag upptäcker problem med läkemedelshanteringen är det svårt att säga ifrån på det här apoteket. (Med läkemedelshantering avses all verksamhet som har med läkemedel att göra, som exempelvis varuhantering och expedition, i både receptur och egenvård).	If I detect problems with medication management, it is difficult to address this, in this pharmacy. (The term "medication management" refers to all activities that have to do with medication, such as product management and expediting of both prescriptions and over-the -counter products).	The original "patient care" was transformed into "handling medications", since patient care is difficult to interpret in this context; in Swedish pharmacies. The statement was also supported by an explanatory sentence, clarifying the definition of "handling medications".

***Safety climate:***			

I would feel safe being treated here as a patient	Jag skulle känna mig trygg som kund här.	I would feel safe as a customer here.	Customer, rather than patient, is the common word in Swedish community pharmacies.

I receive appropriate feedback about my performance.	Jag får konstruktiv återkoppling - feedback - på det arbete jag utför.	I receive constructive feedback for the work I do.	The word "feedback" is sometimes used in Swedish, and it was hence kept but supported by an explanatory Swedish wording.

In this pharmacy, it is difficult to discuss errors.	På det här apoteket är det svårt att diskutera misstag.	In this pharmacy it is difficult to discuss mistakes.	Mistakes include not only errors but also near misses.

***Stress Recognition:***			

Fatigue impairs my performance during emergency situations.	Trötthet försämrar min arbetsinsats i pressade situationer.	Fatigue impairs my performance in trying situations.	Emergency situations are rare in community pharmacies.

***Working conditions:***			

All the necessary information for therapeutic decisions is routinely available to me.	Jag har rutinmässigt tillgång till den information som krävs för att bedöma huruvida en förskrivning är rimlig.	I normally have access to the information needed for judging whether a prescription is reasonable.	The original sentence is not relevant in Swedish pharmacies and had to be rephrased.

One none-scaled item was added. Due to difficulties of interpretation of this item "Communication breakdowns that lead to delays in delivery of medications are common" it was clarified by dividing it into two questions, one focusing on communication breakdown with customer and one focusing on internal communication.

The Swedish Community Pharmacy SAQ questionnaire finally included 40 items (as a result of the alterations mentioned above), of which 30 scaled items are reported in the psychometrics. Only these items, i.e. those used to calculate psychometrics, are included in Additional file 1. Furthermore, the survey contained 19 additional questions regarding the perceptions of safety and teamwork, as well as an open ended item about safe medication handling practices, and demographics. These questions will not be discussed in this paper, because they were not validated. However, the questions were added for latter analyses.

### Survey methodology

A web-based survey methodology was employed for data collection, as it allows a large amount of data to be collected at a relatively low cost. Moreover, the use of web-based surveys is an established and trusted mode of administration within Swedish pharmacies, since employee surveys have been conducted this way annually since 2004 [31]. All pharmacies are computerized and all members of staff have their own e-mail-address.

### Implementation of the survey

The National Corporation of Pharmacies provided protected time for the staff to answer the questionnaire during working hours. The SAQ, together with additional supplemental information was estimated to take approx 15-20 minutes to answer.

In order to further enhance the reliability and internal construct of the questionnaire, it was administered in two waves. A first wave to 66 pharmacies or 1,502 respondents was distributed in a limited roll-out in January 2008. After two weeks, an interim analysis was carried out, covering the first 419 (29.9%) responses received. The psychometric analysis confirmed that the survey was working and the second wave was distributed two weeks later, covering 5,742 respondents. The survey was finally closed in March 2008.

The response-rate was continuously monitored and five web-based reminders were administered. Additional actions that were taken in order to improve the response rate during the study period was direct contact with managers (e-mails, meetings and telephone calls) at various levels within the corporation. The two labour unions organising the majority of staff in the pharmacies, submitted information regarding the study to their members, encouraging them to participate, prior to the start of the study.

### Outcome measures

For ease of interpretation, scale scores were converted from the 5 point scale (1 = Disagree Strongly, 2 = Disagree Slightly, 3 = Neutral, 4 = Agree Slightly, 5 = Agree Strongly) to a 100 point scale in which a score of 100 is "ideal" [20]. All answers were used in the evaluation but only pharmacies with responses from five or more respondents, constituting a minimum of 60% respondents in those settings, were included in the psychometric and benchmarking efforts per pharmacy. The minimum number of five respondents and the minimum threshold of a 60% response rate are used in SAQ administrations to maintain confidentiality of respondents, and to generate reliable consensus safety culture domain scores from people who are working in a particular setting [21]. In order to speak of a culture, the same opinions have to be expressed by a large fraction of the respondents, in this case set at 60%.

The interpretation of the results within a given setting is that if ≥ 80% of the respondents report positive assessments on a specific item or set of items, then there is a strong positive consensus in that setting. In other words, the goal is to have 4 out of 5 respondents report positive assessments [21]. A score of less than 60% is considered to be in the "needs improvement," range.

#### Statistical analyses

Scale psychometrics were calculated including scale reliability and intercorrelations for the scales. Positive SAQ scale scores; ≥ 75 out of 100; i.e. agree slightly or strongly, were calculated and charted per dimension. Reporting only mean values can mask the extent to which an SAQ scale score has a large or small standard deviation, which can vary significantly. Charting the percent of respondents reporting positive scores more precisely assesses the homogeneity of the SAQ constructs within a given pharmacy. Scale reliabilities, specifically internal consistency, of the six scales were assessed using Cronbach's alpha. Multiple group confirmatory factor analysis (CFA) was conducted using AMOS 17.0.

## Results

**Figure 1:**
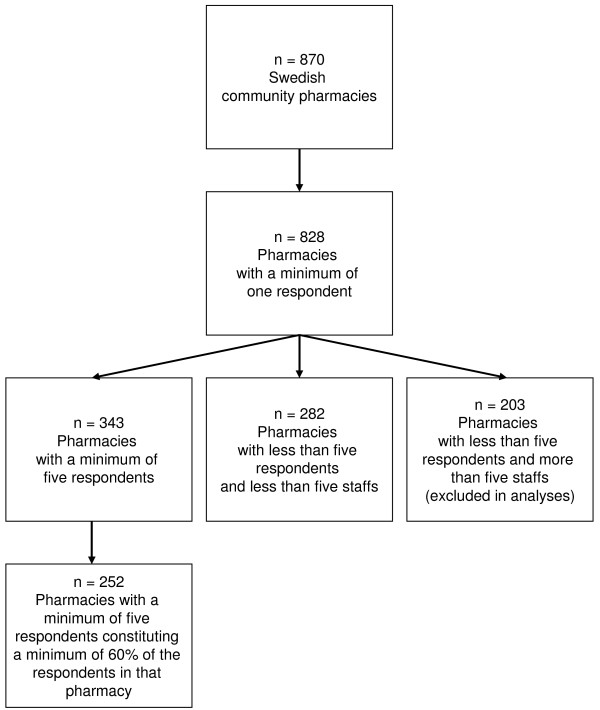
**Swedish Community pharmacies - responses to the SAQ**.

Of all 870 Swedish community pharmacies participating in the web-based survey, data were returned from 828 (95.2%), Figure 1. Of the participating pharmacies 343 units (39.42%) returned data from at least five respondents. 252 pharmacies (28.97%) returned data from at least five respondents, constituting a minimum of 60% of the possible respondents in those pharmacies. These 252 pharmacies represented almost half (45.49%) of the 554 pharmacies that had at least 5 potential respondents. Given that only 554 pharmacies out of 870 were eligible for evaluation, i.e. had five staff members or more, the actual proportion of responding pharmacies with a minimum of 5 respondents was thus 343 out of 554 (61.91%). In addition there were 282 pharmacies, in which there were less than five staff members and these pharmacies were aggregated into one group (Figure 1). Consequently all 828 participating pharmacies were used in at least some part of the psychometric validation.

Data were returned from 4,090 out of 6,683 eligible responders, resulting in an overall response rate of 61.20%. (It was revealed during the administration that out of the original 7,244 distributed questionnaires some reached staff who were employed by regional offices and over-the-counter stores. Only respondents from community pharmacies are included in the results.)

The respondent demographics are provided in Table 3. The majority of the staff had finished their education more than 11 years prior to the survey (68%) and had spent more than 5 years in their present work place (60%).

**Table 3:** Sample Characteristics of Respondents in Swedish SAQ Community pharmacy version

			Gender		Age		
Pharmacy Position	N	%	N	% female	N	Mean	Range
Head of pharmacy/Head of group of pharmacies	102	2.5	102	87	102	52	28 - 65
Pharmacist responsible for quality and patient safety issues*	582	14.2	582	94	582	52	25 - 67
Pharmacist or prescriptionist	2183	53.4	2182	96	2167	49	23 - 69
Pharmacy technician	1042	25.5	1042	98	1011	51	22 - 66
Pharmacy assistant	50	1.2	50	90	47	39	21 - 64
Other	94	2.3	94	90	93	42	21 - 62
***Total***	4053	99.1	4052	96	4021	50	21 - 69
***Missing***	37	0.9	38		69		
**Total**	4090	100	4090		4090		

* Special pharmacy role, one per pharmacy

### Missing data

Missing data at the item level is displayed in Additional file 1. On average, for all items, the internal attrition was 3.1%, with a range of 1.5 to 4.9%. These values were higher than benchmarking data [20]. The item "Dispensing errors are handled in a correct way at this pharmacy" (Safety Climate) provided the highest value (4.9%).

The answering alternative "Not applicable" was on average used in 2.2% of the responses to all items, with a range of 0.1 to 13.8%. All four items in the Working conditions dimension had high proportions of "Not applicable"-answers, ranging from 7.4 to 13.8% (Additional file 1).

### The Six Factors of the Safety Attitudes Questionnaire

Scale means, standard deviations, the proportion of positive scores (≥ 75 out of 100) and alpha values are presented in table 4. Item responses were skewed towards the positive, but showed considerable variation. All factors with the exception of Stress Recognition, displayed higher means than benchmarking data [20].

**Table 4:** Scale Psychometrics

	Scale Descriptives			Reliability; internal consistency
Scale	M	SD	% positive	α
Teamwork Climate	83.20	18.26	78.08	.81
Safety Climate	80.43	15.61	72.33	.75
Job Satisfaction	82.62	20.17	76.49	.89
Perceptions of Management	70.25	21.64	53.58	.72
Stress Recognition	72.15	22.73	66.28	.86
Working Conditions	73.23	20.83	57.91	.72

Means, Standard Deviations, Percent of Positive Scores (≥ 75 out of 100) and Coefficient Alphas. N = 4.090

The psychometric validation of the scale displayed that coefficient alpha value for each of the scales ranged from .72 to .89. Analyzing the reliability in the dimensions Teamwork Climate and Safety Climate, using the positively worded items that supplemented the Swedish version of the SAQ as described in Method, resulted in a slight improvement of the alpha values which were found to be 0.84 and 0.80, respectively.

### Confirmatory factor analysis

The multiple group confirmatory factor analysis was performed on the full sample of 828 pharmacies, the subset of pharmacies meeting the selection criteria of five respondents and a 60% response rate, the subset of pharmacies with 5 respondents and the subset of pharmacies with less than 5 staff members.

The values displayed in table 5, indicate an acceptable fit of the Swedish SAQ model to the dataset, also when analyzing the smaller pharmacy groups [32]. The CFI [33] was above .90 for all groups with the exception of the small pharmacies. RMSEA values are well below .08 and in fact closer to .05, indicating a good fit of the model [34].

**Table 5:** AMOS Goodness of Fit

	Full sample of Swedish Pharmacies (n = 828)	Swedish Pharmacies with at least 5 respondents (n = 343)	Swedish Pharmacies with at least 5 respondents and 60% response rate (n = 252)	Swedish Pharmacies with less than 5 staff (n = 282)	Non-pharmacy data from units using English SAQ (n = 203)
Administered (n)	6,683	4,436	2,759	738	16,184
Completed (n)	4,090	2,923	2,144	738	10,843
Response rate	61.22%	65.89%	77.71%	72.36%	67.00%
Grouping factor	Pharmacy	Pharmacy	Pharmacy	Not grouped	Nursing unit
CFI	.903	.902	.900	.886	.900
RMSEA	.057	057	.059	.060	.050

Note: Non-pharmacy data from Sexton et al., 2006 ^20^

CFI = Comparative Fit Index; RMSEA = Root Mean Square Error of Approximation.

Indices for Confirmatory Factor Analyses of the Six-Factor Model of Safety attitudes

The two negatively worded items from the original English SAQ were modelled using both positive and negative Swedish translations, but this did not significantly change the fit of the model so the translation of the negatively worded items were retained for use in Table 5 to maintain consistency with other applications of the SAQ.

### Comparison of SAQ Factors across 252 pharmacies (2,144 respondents)

Using only the pharmacies meeting the conditions set for evaluation; i.e. at least 5 respondents constituting a minimum of 60% of the respondents in those pharmacies, perceptions of the pharmacy were moderately to highly correlated with one another (i.e. Teamwork Climate, Job Satisfaction, Perceptions of Management, Safety Climate, and Working Conditions) whereas attitudes about stress (Stress Recognition) had only low correlations with other factors (table 6).

**Table 6:** Intercorrelations for the Scales

	Teamwork Climate	Safety Climate	Job Satisfaction	Perceptions of Management	Stress Recognition	Working Conditions
**Teamwork Climate**						
**Safety Climate**	.68; .68					
**Job Satisfaction**	.65; .73	.65; .66				
**Perceptions of Managements**	.49; .42	.57; .51	.58; .55			
**Stress Recognition**	-.08; -0.04	-.12; -0.03 ns	-.13; -0.09 ns	-.15; -0.17		
**Working Conditions**	.49; .53	.58; .58	.54; .54	.63; .60	-0.12; -0.17	

Pearson correlation is used for calculations

Note: All correlations significant at < .01 unless noted as not significant (ns).

Values at the respondent level (n = 2,923) and the pharmacy level (n = 252): respondent level; pharmacy level

**Figure 2:**
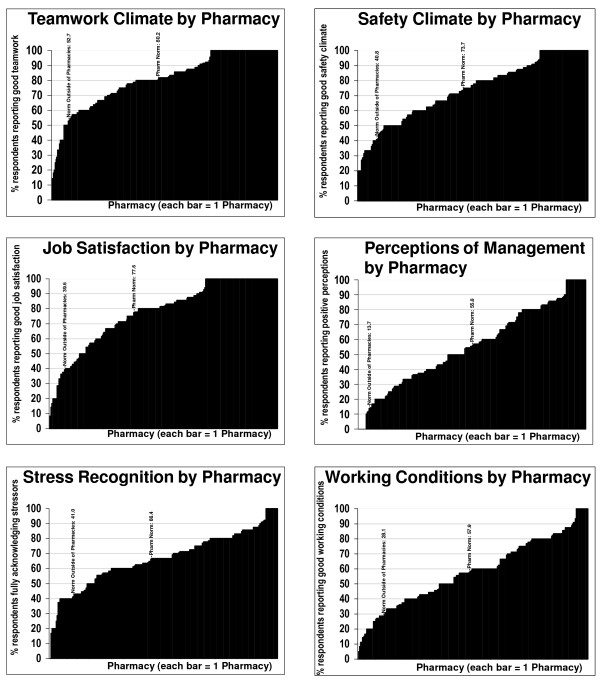
**Distribution of percent positive scores for 252 Swedish Pharmacies**. The figure includes the Swedish Pharmacy norm (the average agreement) and a norm from outside of pharmacy using ICUs, Operating Rooms, Inpatient, and Ambulatory units from Sexton et al. 2006.

The data in Figure 2 provides insight in the variability in different social, psychological and organizational factors pertaining to the safety culture, across Swedish pharmacies. Percents of positive SAQ scale scores (≥ 75 out of 100) were charted to demonstrate the variability across pharmacies in a manner which reflected both the presence and magnitude of each SAQ factor.

There was substantial variability ranging from 0% to 100% in the percent of positive scores for each of the factors across the pharmacies (n = 252). Perceptions of management showed the most variability across pharmacies (SD = 26.66), whereas Stress Recognition showed the least (SD = 18.58). The percent of positive scale scores were generally lower for Perceptions of Management and Working Conditions, relative to the other four factors. In summary, the data in Figure 2 provide a comparative empirical context in which to interpret the factors of importance to the safety culture in a given pharmacy. As compared to the norms from outside pharmacy, the Swedish pharmacies displayed a higher percent of positive scale scores for all factors.

## Discussion

This study demonstrates that the Safety Attitude Questionnaire has been successfully adapted for use in Swedish community pharmacies. The psychometric properties are satisfactory and the existence of comparable data from other settings within the health-care system makes this a valuable tool for use within and between pharmacies. The adaptation of this instrument to the Swedish pharmacy context not only is an important milestone for safety research in pharmacies but also provides the pharmacies with an instrument that generates diagnostic and actionable information for pharmacies and managers to use in guiding improvement efforts [35].

As a measure of consensus, safety culture surveys require a representative number of respondents to ensure confidentiality and reliability of results, but this was a limitation in the current study where 282 responding community pharmacies employed fewer than 5 workers. It is encouraging, however, that the psychometrics of the survey in these 282 pharmacies appeared to be highly consistent with results from pharmacies with 5 or more respondents. Exploring the minimum number or respondents needed to provide a robust safety culture score is a worthwhile direction for future research.

The questionnaire was estimated to require 15-20 minutes completing. Reactions from some respondents implied that this amount of time sometimes was not available at the workplace. This might possibly have impacted the response rate negatively.

The Swedish version of the Safety Attitude Questionnaire used here consisted of 40 items since amendments, as described under Methods, resulted in the addition of items. Altogether the entire survey the respondents received was about twice as long as usual and without the additional questions, an even higher response rate may have resulted.

### Item Response

There was a relatively large proportion of missing data and "not applicable" responses within the dimension Working conditions. Two of the items dealt with the handling of new staff and one explanation might be that the respondents simply did not know the answer, since there had been no change in staff in their pharmacy for a long time. The question "I normally have access to the information needed for judging whether a prescription is reasonable" could have been considered, by parts of the staff, as a special pharmacist question. A rephrasing of these questions should be considered when reproducing this survey. This might also indicate that this dimension is not appropriately covered in the Swedish version of the SAQ.

### Internal Consistency

The internal consistency in this survey was, in all dimensions, found to be relatively high and quite acceptable. The Swedish translation demonstrated good psychometric properties which were highly consistent with comparable benchmarking data ^20^. Similarly, the Confirmatory Factor Analysis conducted on the full sample as well as the subsets demonstrated consistently acceptable fit indices, and was very similar to the fit indices of the original English version. Also following the pattern of the English version, some of the dimensions were moderately to highly correlated to each other. Although this is considered acceptable, it also suggests that further refinement of the Swedish SAQ may be needed, and in particular, exploring the variability in the SQ dimensions as they relate to pharmacy outcomes will be critical.

### Factor response

The Cronbach's alpha values varied from 0.72 to 0.89 for the six factors. The internal consistency was high for the Teamwork Climate dimension as compared to other data [36]. Also, there was substantial variability in the percent of positive scores for each of the factors across 252 Pharmacies. The percent of positive scale scores across Swedish pharmacies were generally lower for Perceptions of Management and Working Conditions. Team Work Climate and Job Satisfaction displayed, on the other hand, relatively high values across Swedish pharmacies. Benchmarking data indicate that this is also the result within other SAQ assessments [20,36], with the exception of the Team Work Climate dimension. This factor demonstrated notably high proportions of positive responses in Swedish pharmacies, as compared to other samples from outside of the pharmacy environment [20,21,24,26-29,37].

The possible explanation of respondents "social desirability" among respondents is is likely to be limited, because there would have been similar "inflated" patterns in the Perceptions of Management domain, for example. Also, the company history of frequent employee surveys have not revealed any response desirability trends of this kind but rather, that the result is likely to reflect the honest assessments of staff [31].

Also, the relatively high proportion of positive responses might be that the translation was inadequate, and that a ceiling effect occurred. Nevertheless, the use of both positively and negatively worded items in the Swedish translation of the teamwork climate items helped to minimize this risk.

Just as Teamwork Climate appeared to be relatively high among these Swedish community pharmacies, a similar result was recently reported in a study of moral distress among pharmacists, such that they were tolerant and well functioning relative to hospital departments [38]. Moreover, the Swedish management culture is relatively non-hierarchical and democratic, by international comparison [39]. It is also considered to be practiced with a collective orientation and reduced hierarchies [40]. Consequently, the results of this current study may reflect a reality of better collaboration, as seen through other metrics, whereby good relationships are a fundamental and a well-established part of the Swedish working life [41].

## Conclusions

Following the results of the national survey of safety culture in Swedish community pharmacies, it can be concluded that the Swedish translation and adaptation of the SAQ demonstrated satisfactory psychometric properties. Further studies, to link the outcome of the SAQ questionnaire with pharmaceutical outcomes variables are important, i.e. to demonstrate if a high safety culture is associated with timely and accurate dispensing processes and customer satisfaction. Identifying factors influencing the safety culture in pharmacies is vital, since it can support decisions about actions to be taken to improve the safety climate at pharmacies.

## Competing interests

Annika Nordén-Hägg was, at the time of planning and data collection, employed by the National Corporation of Swedish Pharmacies.

Sofia Kälvemark Sporrong was, at the time of planning and data collection, employed by the National Corporation of Swedish Pharmacies.

Bryan Sexton reports receiving grant support from the Gottlieb Daimler and Karl Benz Foundation, and the Robert Wood Johnson Foundation (#58292). Pascal Metrics has licensed the rights to the SAQ described in this article. Dr. Sexton is the inventor of the SAQ and although he has not done so yet, he has permission to work as a paid consultant to Pascal Metrics. The terms of this arrangement are being managed by the Johns Hopkins University in accordance with its conflict of interest policies.

## Authors' contributions

ANH - Initiating project, planning project, translation and adaptation of Swedish SAQ version, acquisition of data, analysis and interpretation of data, drafting of manuscript, revising manuscript, final approval. JBS - Translation and adaptation, analysis and interpretation of data, drafting of manuscript, revising manuscript, final approval. SKS - Planning project, translation and adaptation, analysis and interpretation of data, revising manuscript, final approval. LR - Planning project, translation and adaptation, revising manuscript, final approval. AKL - Planning project, translation and adaptation, analysis and interpretation of data, revising manuscript, final approval

## Pre-publication history

The pre-publication history for this paper can be accessed here:

http://www.biomedcentral.com/1472-6904/10/8/prepub

## Supplementary Material

Additional file 1 **Item variation by factor**. Dimensions and items; Proportion of missing, "Not applicable", "Agree" ("Agree Strongly") and "Disagree"("Disagree Strongly") answers and Cronbach's alpha values. Click here for file
